# Molecular detection of tumour DNA in serum and peritoneal fluid from ovarian cancer patients

**DOI:** 10.1038/sj.bjc.6690601

**Published:** 1999-08

**Authors:** K P Hickey, K P Boyle, H M Jepps, A C Andrew, E J Buxton, P A Burns

**Affiliations:** Department of Gynaecological Oncology, Leeds General Infirmary, Leeds, LS1 3EX, UK; Department of Pathological Sciences, Algernon Firth Building, University of Leeds, Leeds, LS2 9JT, UK

**Keywords:** ovarian cancer, molecular detection, microsatellite instability, serum

## Abstract

We have analysed DNA extracted from the serum and peritoneal fluid of 20 ovarian cancer patients for the presence of tumour-specific genetic alterations. The 20 patients included six with stage Ia disease. Using six polymorphic microsatellite loci we were able to detect novel alleles or loss of heterozygosity in 17/20 serum samples and 12/19 peritoneal fluid samples. Tumour-specific abnormalities were detected in the serum of all but one of the stage Ia cases. Half of the occurrences of loss of heterozygosity identified in primary tumour material were detectable in the serum samples. Novel alleles indicative of microsatellite instability were found in 3/6 patients with stage Ia disease but in only 1/14 of patients with more advanced disease. One of the eight patients in the control group displayed abnormalities in her serum DNA. The ease with which tumour-specific alterations were detected in serum and peritoneal samples from ovarian cancer patients, using a panel of only six polymorphic microsatellite markers on four chromosomes, suggests that molecular detection methods could prove useful in the staging, monitoring and screening of this disease. © 1999 Cancer Research Campaign


*British Journal of Cancer* (1999) **80**(11), 1803–1808
© 1999 Cancer Research Campaign
Article no. bjoc.1999.0601

### Molecular detection of tumour DNA in serum and peritoneal fluid from ovarian cancer patients

**KP Hickey[1], KP Boyle[2], HM Jepps[2], AC Andrew[2], EJ Buxton[1] and PA Burns[2]**

[1]Department of Gynaecological Oncology, Leeds General Infirmary, Leeds LS1 3EX, UK; [2]Department of Pathological Sciences, Algernon Firth Building, University of Leeds, Leeds LS2 9JT, UK

**Summary** We have analysed DNA extracted from the serum and peritoneal fluid of 20 ovarian cancer patients for the presence of tumour-specific genetic alterations. The 20 patients included six with stage Ia disease. Using six polymorphic microsatellite loci we were able to detect novel alleles or loss of heterozygosity in 17/20 serum samples and 12/19 peritoneal fluid samples. Tumour-specific abnormalities were detected in the serum of all but one of the stage Ia cases. Half of the occurrences of loss of heterozygosity identified in primary tumour material were detectable in the serum samples. Novel alleles indicative of microsatellite instability were found in 3/6 patients with stage Ia disease but in only 1/14 of patients with more advanced disease. One of the eight patients in the control group displayed abnormalities in her serum DNA. The ease with which tumour-specific alterations were detected in serum and peritoneal samples from ovarian cancer patients, using a panel of only six polymorphic microsatellite markers on four chromosomes, suggests that molecular detection methods could prove useful in the staging, monitoring and screening of this disease.

**Keywords:** ovarian cancer; molecular detection; microsatellite instability; serum

Ovarian cancer is the leading cause of death from gynaecological malignancy in the UK, with over 4000 women dying annually. The high mortality is attributable to the lack of effective screening strategies and the fact that early-stage disease remains clinically silent. Most patients present with advanced disease, for which the long-term survival remains <30%. However, if early stage disease could be detected the 5-year survival could rise to 80–95% (Berek, 1994). Current ovarian cancer screening methods using serum tumour markers and ultrasound/Doppler techniques lack the specificity to achieve this goal at present.

Nucleic acid-based molecular detection methods have been used to demonstrate the presence of tumour cells in samples of stool (Sidransky et al, 1992), urine (Mao et al, 1996), sputum (Mao et al, 1994) and blood (Smith et al, 1991). In addition, the same approaches have confirmed the presence of free circulating tumour DNA in the serum of patients with epithelial tumours of the lung (Chen et al, 1996), head and neck (Nawroz et al, 1996) and colon (Hibi et al, 1998). The majority of these studies have demonstrated clear clonality between the genetic alterations observed in test samples and the corresponding primary tumour material. The types of alteration recorded have included specific gene mutations, loss of heterozygosity (LOH) and microsatellite instability (MI).

Only 5–10% of ovarian carcinomas appear to involve a familial predisposition. The two major syndromes involved are the familial breast and ovarian cancer syndrome, associated with inherited mutations in the BRCAl gene, and the Lynch II syndrome (HNPCC), associated with inherited defects in the DNA mismatch repair system. Abnormalities associated with sporadic forms of the disease include mutations in k-*ras* and *p53* genes, and changes in expression of *HER-2* (Gallion et al, 1995). Allelotyping studies have identified common sites of LOH on chromosomes 5q, 6q, 11p, 13q, 14q, 15q, 18q, Xp (Cliby et al, 1993; Osborne and Leech, 1994), and particularly on chromosome 7q (Zenklusen et al, 1995), 9q (Schultz et al, 1995) and 17p (Phillips et al, 1993). The occurrence of novel alleles at microsatellite loci, due to either a replication error phenotype ($RER^+$) resulting from defective mismatch repair or general genetic instability, has been described in 17–37% of ovarian tumours (Fujita et al, 1995; King et al, 1995; Sood and Buller, 1996). However, MI may occur in up to 75% of stage I ovarian tumours, and 71% of uncommon histopathological types such as endometrioid or mixed serous and mucinous tumours (King et al, 1995).

We obtained serum and peritoneal fluid from 20 women with epithelial ovarian carcinoma, and used a panel of six polymorphic microsatellite markers to detect LOH or novel alleles in the DNA extracted from the samples. The same analysis was carried out on tumour tissue microdissected from paraffin sections of the primary tumour from each case. The aim of this study was to establish whether tumour-specific biomarkers could be detected in the serum and peritoneal fluid of ovarian cancer patients, particularly those with early-stage disease.

*Received 25 September 1998*
*Revised 28 January 1999*
*Accepted 4 February 1999*

*Correspondence to:* PA Burns

### MATERIALS AND METHODS

### Sample collection

Samples were collected from 20 women undergoing laparotomy for ovarian cancer, and eight women with benign or physiological cysts. None of the women were known to carry a familial

**Table 1** Staging, pathology, cytology and genetic analysis of ovarian cancer patients

| Patient | Stage[a] | Histology type and differentiation | Cytology[b] | Tumour | | Serum | | Peritoneal fluid | |
|---|---|---|---|---|---|---|---|---|---|
| | | | | LOH[c] | MI[d] | LOH | MI | LOH | MI |
| 112 | Ia | Endometrioid, Grade 1 | – | 1/6 | – | 2/6 | – | 0/6 | – |
| 200 | Ia | Endometrioid and mucinous, Grade 2 | – | 1/5 | – | 0/5 | – | 0/5 | – |
| 3775 | Ia | Mucinous, Grade 2 | – | 0/1 | 5 | 0/4 | 1 | 0/4 | 1 |
| 4224 | Ia | Mucinous, Grade 2 | – | 0/3 | 2 | 0/3 | 2 | 0/3 | 2 |
| 5475 | Ia | Serous, Grade 2 | – | 3/4 | – | 2/4 | – | 0/4 | – |
| 065 | Ia | Endometrioid, Grade 1 | – | 1/3 | 1 | 2/3 | 1 | NA | – |
| 705 | IIc | Serous, Grade 3 | + | 1/5 | – | 1/5 | – | 1/4 | – |
| 488 | IIIc | Serous, Grade 3 | + | 2/3 | – | 2/3 | – | 2/3 | – |
| 165 | IIIc | Papillary serous, Grade 2 | – | 3/3 | – | 3/3 | – | 1/3 | – |
| 231 | IIIc | Papillary serous, Grade 2 | – | 4/5 | – | 2/5 | – | 1/5 | – |
| 042 | IIIc | Serous, Grade 3 | – | 5/5 | 1 | 2/5 | – | 0/5 | – |
| 285 | IIIc | Papillary serous, Grade 3 | – | 3/4 | – | 1/4 | – | 1/4 | – |
| 577 | IIIc | Endometrioid & serous, Grade 3 | + | 0/4 | – | 2/4 | – | 1/4 | – |
| 0776 | IIIc | Papillary serous, Grade 3 | + | 0/3 | – | 1/3 | – | 0/3 | – |
| 9930 | IIIc | Serous, Grade 3 | – | 5/5 | – | 3/5 | – | 5/5 | – |
| 4992 | IIIc | Papillary serous, Grade 3 | – | 2/4 | – | 2/4 | – | 1/4 | – |
| 5042 | IIIc | Endometrioid, Grade 3 | + | 2/5 | – | 0/5 | – | 0/5 | – |
| 3944 | IIIc | Serous, Grade 3 | – | 0/4 | – | 1/4 | – | 0/4 | – |
| 4571 | IIIc | Papillary serous, Grade 3 | + | 3/4 | – | 1/4 | – | 2/3 | – |
| 421 | IV | Endometrioid, Grade 3 | + | 3/4 | – | 0/4 | – | 3/4 | – |
| | | | Total | 39/80 | 9 | 27/83 | 4 | 18/78 | 3 |

[a] FIGO staging of primary tumour; [b]presence of malignant cells in ascitic fluid or peritoneal washings; [c]proportion of informative (heterozygous) markers displaying loss of heterozygosity (LOH); [d]microsatellite instability. NA, not available for molecular analysis.

predisposition for ovarian cancer, although one woman had a first-degree relative with breast cancer. The cancer cases included six stage Ia and 14 stage II–IV cases. In all six of the stage Ia cases total abdominal hysterectomy, bilateral salpingo-oopherectomy, omentectomy and peritoneal washings for cytology were taken, while ipsilateral pelvic node resection and para-aortic node sampling was carried out in three of the six. The pathological staging, serum CA125 and cytology results are shown in Table 1.

### DNA extraction

### *Normal DNA*

Ten millilitres of blood were taken into heparinized tubes, and 1 ml was used for extraction of white blood cell DNA using Nucleon (Scotlab).

### *Serum DNA*

Ten millilitres of blood was taken, allowed to clot and then centrifuged at 1300 ***g***. The serum supernatant was frozen at –80°C in aliquots and DNA was extracted from 400 μl using DNAzol (Gibco BRL).

### *Peritoneal fluid DNA*

At laparotomy, 20 ml of ascitic fluid or pelvic peritoneal washings were taken, of which 10 ml were sent for routine cytological analysis, and the other 10 ml for molecular analysis. The latter samples were centrifuged at 1300 ***g*** to pellet any cells, which were washed twice in phosphate-buffered saline (PBS). The cells were frozen at –80°C in tissue culture-freezing medium (2.75 ml Dulbecco's modified Eagle's medium, 1.25 ml fetal calf serum, 1 ml dimethyl sulphoxide (DMSO)) in 1 ml aliquots. Cells were spun out of thawed aliquots and washed twice with 5 ml PBS to remove DMSO. Epithelial cells were then harvested by immuno-magnetic separation using Dynabeads coated with the epithelial specific antibody Ber EP4 (Dynal UK). DNA was extracted from the cells using Dynabeads DNA Direct (Dynal UK). A peritoneal fluid sample from patient 065 was unavailable for molecular analysis.

### *Tumour DNA*

Areas of tumour tissue were microdissected from ten paraffin sections of primary tumours and DNA extracted using Nucleon (Scotlab). The precipitated DNA was resuspended in 50 μl of water.

### *Cyst wall DNA*

For cases of benign and physiological cyst, areas corresponding to the cyst wall were microdissected and processed in the same way as the tumour DNA.

### Microsatellite analysis

Polymerase chain reaction (PCR) amplification was carried out using oligonucleotide amplimers specific for six polymorphic DNA microsatellite loci:

DP1 (D5S346) on 5q (Spirio et al, 1993)
486 (D7S486) on 7q (Gyapay et al, 1994)
522 (D7S522) on 7q (Gyapay et al, 1994)
D11 (D11S904) on 11p (Gyapay et al, 1994)
p53V (intron1 in p53) on 17p (Cawkwell et al, 1994)
BRCA1 (D17S855) on 17q (Gao et al, 1995).

The chosen markers map to regions which commonly display LOH in ovarian carcinomas. One amplimer from each pair was fluorescently labelled using the dye-amidite method (Cawkwell et al, 1994).

The PCR reactions were set up in 25 μl of 1 × Super*Taq* Reaction Buffer (HT Biotechnology Ltd) containing 200 μM dNTPs, 12.5 pmol of each amplimer, 1.5–6.0 mM magnesium

*© 1999 Cancer Research Campaign*

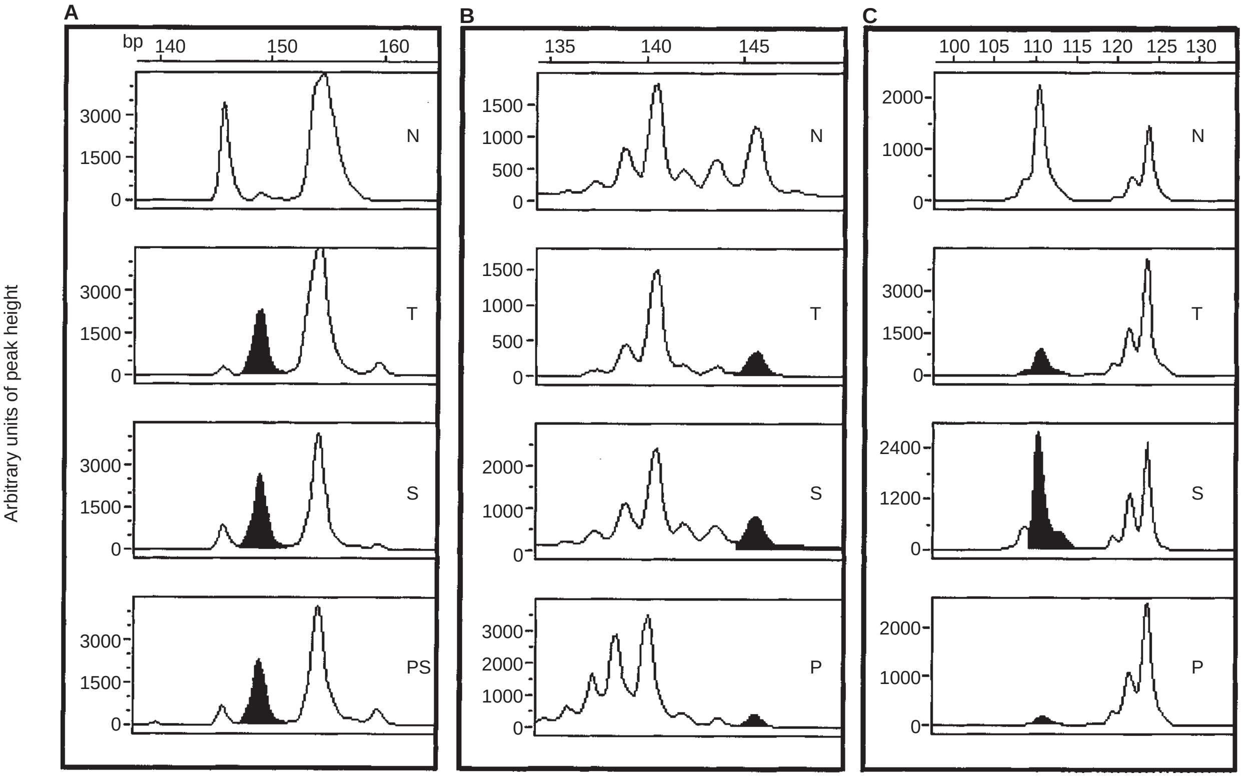

**Figure 1** Electropherograms of microsatellite instability (MI) or loss of heterozygosity (LOH) in three ovarian cancer patients. Fluorescently labelled PCR products were run on an ABI 373A DNA Sequencer. The fluorescent signals were converted into electropherograms by Genescan Software. Length of product in base pairs is indicated on the X-axis and an arbitrary measurement of peak height, proportional to signal intensity, on the Y-axis. (**A**) MI at p53V locus seen in patient 4224; (**B**) LOH at the D7S486 locus seen in patient 165; (**C**) LOH at the D5S346 (DP1) locus seen in patient 4571. N, normal DNA; T, tumour DNA; S, serum DNA; P, peritoneal fluid DNA

chloride (optimized for each pair of amplimers), and 1 μl of DNA. After denaturing at 95°C for 3 min and a pause at 80°C to add 0.5 U of Super*Taq* polymerase (HT Biotechnology Ltd), the DNA was amplified using a programme of 92°C for 30 s, 56°C for 1 min, 72°C for 30 s, for a total of 35 cycles. The amplification products were visualized by agarose gel electrophoresis to estimate yield, electrophoresed on an ABI 373A DNA Sequencer (Applied Biosystems), and analysed using Genescan Analysis Software (Applied Biosystems), which determines the size of the PCR products and the amount of fluorescent signal. Suitable dilutions of PCR products were run to ensure that band intensities fell within the linear portion of the fluorescence detection range (500–4000).

### Optimizing template DNA for PCR

We found that significantly different allele ratios could be obtained in our PCR reactions with different amounts of starting template DNA. It is difficult to accurately quantify the amount of DNA actually available for PCR amplification in a small sample. It was therefore essential to use different dilutions of normal control DNA (down to $1 \times 10^{-4}$) and choose the dilution which produced a comparable yield, as estimated by agarose gel electrophoresis, to that of the tumour/serum/peritoneal fluid samples as the control lane for Genescan analysis.

### LOH and MI

The use of Genescan software and multiple PCR runs allowed us to accurately measure alterations in allele ratios. LOH was defined as a >20% shift in allele ratios (see below), as calculated using the equation $T_1{:}T_2/N_1{:}N_2$ (where $T_1{:}T_2$ is the ratio of the two tumour alleles and $N_1{:}N_2$ is the ratio of the corresponding normal alleles). Novel alleles were defined as one or more novel peaks consistent in size with expansion or contraction of the microsatellite repeat and distinct from PCR stutter products. All LOHs or MIs were confirmed by multiple independent PCR amplifications.

### RESULTS

### Defining parameters for LOH

An important consideration in studies which look for LOH in tumour DNA is deciding what constitutes a significant shift in allele ratio at heterozygous loci. The use of Genescan Analysis software allowed us to accurately compare allele ratios from tumour, peritoneal fluid and serum DNA samples, with those obtained from normal DNA. Analysis of over 250 of these comparisons revealed a clear bimodal distribution, with 99% of values falling between 0 and 80% or 85 and 100% (data not shown), with a striking gap between 80 and 85%. On this basis we assumed that an allele ratio shift of 0–15% represented normal variation in the amplification of alleles present in equimolar amounts in normal diploid DNA, whereas a shift of >20% represented a notable deviation from a balanced allele ratio. For the purposes of this study we therefore chose a >20% shift as the criteria for defining LOH in our samples. However, most allelotyping studies using microdissected paraffin-embedded tumour material tend to use a >50% shift to define LOH. We therefore also

© 1999 Cancer Research Campaign 

**Table 2** Results of Genescan analysis of PCR products from 20 ovarian cancer patients analysed for LOH or microsatellite instability at six microsatellite loci

| Patient | Sample | DP1 | 522 | 486 | D11 | P53 | BRCA1 |
|---|---|---|---|---|---|---|---|
| 112 | Tumour | ○ | ○ | ○ | ● | ○ | ○ |
| | Serum | ○ | ○ | ○ | ● | ● | ○ |
| | Per. fluid | ○ | ○ | ○ | ○ | ○ | ○ |
| 200 | Tumour | ○ | ○ | ○ | ○ | × | ● |
| | Serum | ○ | ○ | ○ | ○ | × | ○ |
| | Per. fluid | ○ | ○ | ○ | ○ | × | ○ |
| 3775 | Tumour | MI | MI | MI | MI | MI | ○ |
| | Serum | ○ | ○ | ○ | MI | × | ○ |
| | Per. fluid | ○ | ○ | ○ | MI | × | ○ |
| 4224 | Tumour | ○ | × | ○ | MI | MI | ○ |
| | Serum | ○ | × | ○ | MI | MI | ○ |
| | Per. fluid | ○ | × | ○ | MI | MI | ○ |
| 5475 | Tumour | ● | × | ○ | ● | ● | × |
| | Serum | ● | × | ○ | ○ | ● | × |
| | Per. fluid | ○ | × | ○ | ○ | ○ | × |
| 065* | Tumour | ● | MI | × | ○ | ○ | × |
| | Serum | ● | MI | × | ● | ○ | × |
| 705 | Tumour | ○ | ○ | ○ | ○ | × | ● |
| | Serum | ○ | ○ | ● | ○ | × | ○ |
| | Per. fluid | NP | ○ | ○ | ○ | × | ● |
| 488 | Tumour | × | × | ○ | ● | × | ● |
| | Serum | × | × | ○ | ● | × | ● |
| | Per. fluid | × | × | ○ | ● | × | ● |
| 165 | Tumour | × | ● | ● | × | ● | × |
| | Serum | × | ● | ● | × | ● | × |
| | Per. fluid | × | ○ | ● | × | ○ | × |
| 231 | Tumour | ● | × | ○ | ● | ● | ● |
| | Serum | ● | × | ○ | ○ | ○ | ● |
| | Per. fluid | ○ | × | ○ | ○ | ○ | ● |
| 042 | Tumour | ● | ● | ● | ● | MI | ● |
| | Serum | ● | ○ | ○ | ● | × | ○ |
| | Per. fluid | ○ | ○ | ○ | ○ | × | ○ |
| 285 | Tumour | ○ | × | ● | ● | × | ● |
| | Serum | ○ | × | ○ | ○ | × | ● |
| | Per. fluid | ○ | × | ○ | ○ | × | ● |
| 577 | Tumour | ○ | × | × | ○ | ○ | ○ |
| | Serum | ○ | × | × | ● | ○ | ● |
| | Per. fluid | ○ | × | × | ● | ○ | ○ |
| 0776 | Tumour | ○ | × | ○ | ○ | × | × |
| | Serum | ○ | × | ○ | ● | × | × |
| | Per. fluid | ○ | × | ○ | ○ | × | × |
| 9930 | Tumour | ● | ● | ● | ● | × | ● |
| | Serum | ● | ● | ● | ○ | × | ○ |
| | Per. fluid | ● | ● | ● | ● | × | ● |
| 4992 | Tumour | ● | × | × | ● | ○ | ○ |
| | Serum | ● | × | × | ○ | ● | ○ |
| | Per. fluid | ● | × | × | ○ | ○ | ○ |
| 5042 | Tumour | ● | ○ | ○ | ○ | × | ● |
| | Serum | ○ | ○ | ○ | ○ | × | ○ |
| | Per. fluid | ○ | ○ | ○ | ○ | × | ○ |
| 3944 | Tumour | × | ○ | ○ | ○ | × | ○ |
| | Serum | × | ○ | ● | ○ | × | ○ |
| | Per. fluid | × | ○ | ○ | ○ | × | ○ |
| 4571 | Tumour | ● | × | ● | ○ | × | ● |
| | Serum | ● | × | ○ | ○ | × | ○ |
| | Per. fluid | ● | × | ○ | NP | × | ● |
| 421 | Tumour | ● | ○ | × | ● | × | ● |
| | Serum | ○ | ○ | × | ○ | × | ○ |
| | Per. fluid | ● | ○ | × | ● | × | ● |

○ – heterozygosity retained; ● – loss of heterozygosity; MI – microsatellite instability; × – non-informative; NP – no product was obtained from repeated PCR amplifications. *No peritoneal fluid sample was available for molecular analysis from this patient. Per. fluid, peritoneal fluid analysed our results using a >50% shift in allele ratio as the criteria for LOH (see below).

### LOH and MI in primary tumour

All of the primary tumours displayed at least one genetic alteration (Figure 1A–C), with the exception of patients 577, 0776 and 3944 (Table 1). LOH was detected at 39/80 (49%) informative markers, and was twice as common in stage II–IV (33/58, 57%) as in stage Ia tumours (6/22, 27%). LOH occurred most frequently at BRCA1 on chromosome 17q (63%), DP1 on 5q (56%) and D11 on 11p (53%) (Table 2).

Novel alleles were observed in the primary tumours of 3/6 stage Ia patients (3775, 4244, 065), but only in 1/14 patients (042) with more advanced disease (Tables 1 and 2). In the case of patients 3775 (novel alleles at 5/6 loci) and 4224 (2/6) this may be evidence of an $RER^{+}$ phenotype associated with mismatch repair deficiency.

### LOH and MI in serum DNA

Genetic alterations were detected in 17/20 serum DNA samples, including 5/6 of the samples from patients with stage Ia disease (Table 1). LOH was harder to detect in serum (27/83 or 33% of markers) than in the primary tumours (49%). Of the 27 cases of LOH detected in serum, 19 (73%) were observed in the corresponding primary tumour (Figure 1B,C) and eight were specific to the serum DNA (Table 2). Thus 19/39 (49%) examples of LOH found in primary tumours were detectable in serum DNA.

Novel alleles were harder to detect in serum DNA than in primary tumour DNA (Table 1). Of the nine examples found in primary tumours, four were detected in the corresponding serum samples (Table 2 and Figure 1A). The three patients with novel alleles in their serum DNA all had stage Ia disease (Table 1).

### LOH and MI in peritoneal fluid DNA

Only 12 of the 19 DNA samples extracted from peritoneal fluid exhibited genetic alterations, and only 18/78 (23%) heterozygous markers displayed LOH compared to 33% in the serum samples and 49% in the primary tumours (Table 1). However, 17 of the 18 examples of LOH in the peritoneal fluid samples were also found in the corresponding primary tumour (Figure 1B,C), indicating a higher degree of clonality than was seen with the serum samples (Table 2).

Novel alleles were detected in peritoneal fluid DNA from two of the stage 1a patients, all of which had been observed in the primary tumour and serum from the same patients (Table 2 and Figure 1A). By definition, these two patients had negative peritoneal fluid cytology.

### Reanalysis of results using >50% in allele ratios

Out of 84 examples of LOH featured in Table 1, 60 displayed >50% shift in allele ratio. Of the remaining 24, which showed a 20–50% shift, 19 coincided with the finding of a >50% shift in another sample from the same patient. For example, the serum sample from patient 4571 showed a 30% reduction in the shorter allele at marker DP1 (Figure 1C), whereas the tumour and peritoneal fluid samples showed almost complete loss. These observations of significant ($P < 0.001$) clonal similarity support our initial

 *© 1999 Cancer Research Campaign*

assumption that a >20% shift represents a genuine case of LOH. However, taking a >50% shift as the criteria for LOH we would still have detected tumour-specific alterations in 5/6 serum samples from stage Ia patients but only 7/14 stage II–IV patients. In the case of peritoneal fluid we would still have detected alterations in 2/5 stage Ia samples, and 8/14 stage II–IV samples (reduced from 10/14).

### LOH and MI in control cases

DNA was extracted from serum, peritoneal fluid and paraffin-embedded cyst wall samples from four patients with benign mucinous cystadenoma of the ovary and four with physiological ovarian corpus luteal cysts, as controls to evaluate the level of false positive results using this approach. The three samples from each of eight patients were analysed for alterations at all six loci by comparison with normal DNA extracted from white blood cells. Out of a total matrix of 144 genetic analyses, only two displayed genetic abnormalities; an MI at p53 and an LOH at 522, in the serum of a patient with a physiological cyst (results not shown).

### DISCUSSION

We were able to detect genetic alterations in the free circulating DNA extracted from the serum of 17/20 (85%) patients with ovarian carcinoma, using a panel of six microsatellite loci. In addition, exfoliated cells from 12/19 (63%) peritoneal fluid samples were found to contain tumour-specific alterations. For such a limited panel of genetic markers this represents a relatively high degree of sensitivity. Given that all tumour cell populations will contain genetic alterations of some description it should be possible to approach 100% sensitivity using an optimized panel of genetic markers.

Significantly, it was possible to detect tumour-specific genetic markers in serum samples from five out of six patients with stage Ia disease. This suggests that detectable amounts of DNA are released into the blood from tumours confined to one ovary. Previous studies have shown that tumour DNA can be detected in the serum of patients with advanced head and neck (Nawroz et al, 1996), lung (Chen et al, 1996) and colon cancer (Hibi et al, 1998). Our results clearly demonstrate that the earliest defined stage of ovarian carcinoma is detectable using this approach.

Surprisingly, one of the control patients with a physiological cyst had two genetic alterations in her serum DNA which were not present in her peritoneal fluid or ovarian tissue. Although there was nothing in this patient's clinical findings to suggest malignancy, it is feasible that she may have had an occult neoplasm. Genetic alterations of this type should be highly specific for tumour cell populations. If the possibility of PCR artefacts are eliminated, molecular detection techniques used for screening purposes should theoretically approach 100% specificity.

It is not possible to identify the precise biological or physical pathways through which DNA from tumour cells can enter the blood. Tumour cell death can occur through apoptosis, senescence, necrosis or immunosurveillance, any of which could theoretically result in tumour DNA entering the blood stream. The relative contribution of these processes will vary from tumour to tumour, depending on the phenotype of the cells. For example, it has been suggested that the MI/RER$^+$ genotype may make cells more immunogenic, due to accumulation of amino acid alterations in cell antigens, and therefore better targets for immunosurveillance (Bicknell et al, 1996). This could explain the improved prognosis for colorectal cancer patients with this genotype (Lothe et al, 1993; Lukish et al, 1998). In the case of stage Ia ovarian carcinomas, which are by definition confined to one ovary, DNA is probably released directly into the blood stream. With more advanced disease, exfoliated cells in the peritoneal cavity could provide another route of entry of DNA into the blood stream via the lymphatic system.

Our study demonstrates a strong degree of clonality between serum, exfoliated peritoneal cells and primary tumour DNA. Nineteen of 26 and 17/18 alterations seen in the serum and peritoneal fluid, respectively, were also seen in the corresponding primary tumour (Figure 1A–C). However, similar studies in head and neck, lung and colon tumours show almost exclusive clonality (Chen et al, 1996; Nawroz et al, 1996; Hibi et al, 1998). In our study we analysed DNA from a single primary tumour block, usually one showing a preponderance of tumour tissue. Our results would suggest that in 27% of cases the tumour DNA in the serum originates from a subpopulation of cells not significantly represented in the sample microdissected from the primary tumour paraffin block. Such discrepancies may result from tumour heterogeneity and limitations in primary tumour sampling. Another possibility is that clonal subpopulations of cells carrying specific changes may undergo preferential cell death and hence be relatively underrepresented in primary ovarian tumours but enriched for in serum DNA. Previous studies on clonality of ovarian tumours have not addressed genetic heterogeneity within primary tumours.

We find a higher frequency of novel alleles in patients with stage Ia ovarian tumours (3/6) than with stage II–IV (1/14) (Table 1). This is in agreement with an earlier study by King et al (1995) who reported that 75% of stage I tumours had MI compared to 11% of stage II–IV tumours. In addition, LOH is less apparent in patients with stage Ia tumours (27%) compared to stage II–IV (57%). This suggests that stage Ia ovarian tumours are more likely to have an RER$^+$ phenotype than more advanced tumours. Studies on colorectal carcinoma show that patients with RER$^+$ tumours have a better prognosis, because of reduced survival for cells with MI (Lothe et al, 1993; Lukish et al, 1998). In an analogous way the presence of DNA carrying novel alleles in serum may indicate higher levels of tumour cell death and signal a better prognosis for stage Ia ovarian cancer patients.

Two patients with stage Ia disease (3775 and 4224) could technically be upstaged to stage Ic as a result of tumour DNA being detected in their peritoneal fluid. These patients had been diagnosed as stage Ia on the basis of a negative peritoneal fluid cytology result and confinement of disease to one ovary histopathologically. Accurate staging has a significant bearing not only on prognosis, but also on the possible use of adjuvant chemotherapy in this patient subgroup. In the case of head and neck cancer, a prospective multicentre trial is currently underway in the USA to evaluate the efficacy of molecular staging and its impact on surgical practice (Caldas, 1997). To our knowledge, this is the first report of the possible molecular staging of ovarian cancer.

At present, population-based screening for epithelial ovarian cancer is not feasible due to the relatively low prevalence (15/100 000), the lack of specificity of available screening techniques, the absence of a definitive precursor lesion, and the inability to determine a lag time for the development of widespread disease. Advances in transvaginal sonography and colour Doppler imaging

© 1999 Cancer Research Campaign

(van Nagell et al, 1995), as well as the use of serial CA125 measurements (Skates et al, 1995) and complimentary serum markers such as OVX1 (Woolas et al, 1993), have improved early tumour detection. However, the efficacy of periodic screening of even high-risk patients has not been clearly established (Droegemueller, 1994). As molecular detection of tumour DNA in serum or peritoneal fluid seems possible even in stage Ia disease, this technique may hold considerable promise as a secondary screening tool after initial tumour marker or ultrasound abnormalities have suggested ovarian pathology. In terms of disease monitoring it will be interesting to observe if and when tumour DNA in the serum of these patients disappears after treatment, and to what extent its reappearance in serum predates clinical and biochemical evidence of recurrence.

### ACKNOWLEDGEMENTS

We would like to thank Debra Cross and Margaret Longfellow for technical assistance. PB is supported by the Special Trustees of Leeds General Infirmary.

### REFERENCES

Berek JS (1994) Epithelial ovarian cancer. In: *Practical Gynaecological Oncology*, 2nd edn, Berek JS, Hacker NF (eds), pp. 327–375. Williams and Wilkins: Baltimore

Bicknell DC, Kaklamanis L, Hampson R, Bodmer WF and Karran P (1996) Selection for beta 2-microglobulin mutation in mismatch repair-defective colorectal carcinomas. *Curr Biol* **6**: 1695–1697

Caldas C (1997) Molecular staging of cancer: is it time? *Lancet* **350**: 231

Cawkwell L, Lewis FA and Quirke P (1994) Frequency of allele loss of DCC, p53, RBI, WT1, NF1, NM23 and APC/MCC in colorectal cancer assayed by fluorescent multiplex polymerase chain reaction. *Br J Cancer* **70**: 813–818

Chen XQ, Stroun M, Magnenat JL, Nicod LP, Kurt AM, Lyautey J, Lederrey C and Anker P (1996) Microsatellite alterations in plasma DNA of small cell lung cancer patients. *Nat Med* **2**: 1033–1035

Cliby W, Ritland S, Hartmann L, Dodson M, Halling KC, Keeney G, Podratz KC and Jenkins RB (1993) Human epithelial ovarian cancer allelotype. *Cancer Res* **53**: 2393–2398

Droegemueller W (1994) Screening for ovarian carcinoma: hopeful and wishful thinking. *Am J Obstet Gynecol* **170**: 1095–1098

Fujita M, Enomoto T, Yoshino K, Nomura T, Buzard GS, Inoue M and Okudaira Y (1995) Microsatellite instability and alterations in the hMSH2 gene in human ovarian cancer. *Int J Cancer* **64**: 361–366

Gallion HH, Pieretti M, DePriest PD and van Nagell JR (1995) The molecular basis of ovarian cancer. *Cancer* **76**: 1992–1997

Gao X, Zacharek A, Salkowski A, Grignon DJ, Sakr W, Porter AT and Honn KV (1995) Loss of heterozygosity of the BRCA1 and other loci on chromosome 17q in human prostate cancer. *Cancer Res* **55**: 1002–1005

Gyapay G, Morissette J, Vignal A, Dib C, Fizames C, Millasseau P, Marc S, Bernardi G, Lathrop M and Weissenbach J (1994) The 1993–94 Genethon human genetic linkage map. *Nat Genet* **7**: 246–339

Hibi K, Robinson CR, Booker S, Wu L, Hamilton SR, Sidransky D and Jen J (1998) Molecular detection of genetic alterations in the serum of colorectal cancer patients. *Cancer Res* **58**: 1405–1407

King BL, Carcangiu ML, Carter D, Kiechle M, Pfisterer J, Pfleiderer A and Kacinski BM (1995) Microsatellite instability in ovarian neoplasms. *Br J Cancer* **72**: 376–382

Lothe RA, Peltomaki P, Meling GI, Aaltonen LA, Nystrom-Lahti M, Pylkkanen L, Heimdal K, Andersen TI, Moller P, Rognum TO, et al. (1993) Genomic instability in colorectal cancer: relationship to clinicopathological variables and family history. *Cancer Res* **53**: 5849–5852

Lukish JR, Muro K, DeNobile J, Katz R, Williams J, Cruess DF, Drucker W, Kirsch I and Hamilton SR (1998) Prognostic significance of DNA replication errors in young patients with colorectal cancer. *Ann Surg* **227**: 51–56

Mao L, Hruban RH, Boyle JO, Tockman M and Sidransky D (1994) Detection of oncogene mutations in sputum precedes diagnosis of lung cancer. *Cancer Res* **54**: 1634–1637

Mao L, Schoenberg MP, Scicchitano M, Erozan YS, Merlo A, Schwab D and Sidransky D (1996) Molecular detection of primary bladder cancer by microsatellite analysis. *Science* **271**: 659–662

Nawroz H, Koch W, Anker P, Stroun M and Sidransky D (1996) Microsatellite alterations in serum DNA of head and neck cancer patients. *Nat Med* **2**: 1035–1037

Osborne RJ and Leech V (1994) Polymerase chain reaction allelotyping of human ovarian cancer. *Br J Cancer* **69**: 429–438

Phillips N, Ziegler M, Saha B and Xynos F (1993) Allelic loss on chromosome 17 in human ovarian cancer. *Int J Cancer* **54**: 85–91

Schultz DC, Vanderveer L, Buetow KH, Boente MP, Ozols RF, Hamilton TC and Godwin AK (1995) Characterization of chromosome 9 in human ovarian neoplasia identifies frequent genetic imbalance on 9q and rare alterations involving 9p, including CDKN2. *Cancer Res* **55**: 2150–2157

Sidransky D, Tokino T, Hamilton SR, Kinzler KW, Levin B, Frost P and Vogelstein B (1992) Identification of ras oncogene mutations in the stool of patients with curable colorectal tumors. *Science* **256**: 102–105

Skates SJ, Xu FJ, Yu YH, Sjovall K, Einhorn N, Chang YC, Bast RC and Knapp RC (1995) Toward an optimal algorithm for ovarian-cancer screening with longitudinal tumor-markers. *Cancer* **76**: 2004–2010

Smith B, Selby P, Southgate J, Pittman K, Bradley C and Blair GE (1991) Detection of melanoma cells in peripheral blood by means of reverse transcriptase and polymerase chain reaction. *Lancet* **338**: 1227–1229

Sood AK and Buller RE (1996) Genomic instability in ovarian cancer: a reassessment using an arbitrarily primed polymerase chain reaction. *Oncogene* **13**: 2499–2504

Spirio L, Nelson L, Ward K, Burt R, White R and Leppert M (1993) A CA-repeat polymorphism close to the adenomatous polyposis coli (APC) gene offers improved diagnostic testing for familial APC. *Am J Hum Genet* **52**: 286–296

van Nagell JR, Jr, Gallion HH, Pavlik EJ and DePriest PD (1995) Ovarian cancer screening. *Cancer* **76**: 2086–2091

Woolas RP, Xu FJ, Jacobs IJ, Yu YH, Daly L, Berchuck A, Soper JT, Clarke-Pearson DL, Oram DH and Bast RC (1993) Elevation of multiple serum markers in patients with stage I ovarian cancer. *J Natl Cancer Inst* **85**: 1748–1751

Zenklusen JC, Weitzel JN, Ball HG and Conti CJ (1995) Allelic loss at 7q31.1 in human primary ovarian carcinomas suggests the existence of a tumor-suppressor gene. *Oncogene* **11**: 359–363

 *© 1999 Cancer Research Campaign*
